# Targeting neutrophil extracellular traps in bacterial infections: mechanistic insights and translational perspectives

**DOI:** 10.3389/fimmu.2026.1922248

**Published:** 2026-08-28

**Authors:** Shwetha Susan Thomas, Geetha B. Kumar, Bipin G. Nair, Pradeesh Babu, Lekshmi K. Edison, Agiesh Kumar Balakrishna Pillai, Aravind Madhavan

**Affiliations:** 1 School of Biotechnology, Amrita Vishwa Vidyapeetham, Kollam, Kerala, India; 2 Department of Comparative, Diagnostic, and Population Medicine, College of Veterinary Medicine, University of Florida, Gainesville, FL, United States; 3 Department of Clinical Virology, Institute of Advanced Virology, Thiruvananthapuram, Kerala, India

**Keywords:** cell death, host-directed therapy, host-pathogen interactions, neutrophil extracellular trap, neutrophils, sepsis

## Abstract

Neutrophil Extracellular Traps (NETs) are web-like structures composed of DNA fibers, histones, and granular proteins released by the neutrophils activated upon various stimuli. Recognized as a host immune defense mechanism, NETs also play a significant role in several diseases, especially in bacterial infections. Research indicates that excessive NET formation contributes to various pathological outcomes, including multiple organ dysfunction. Once activated, NETs generally remain in a hyperinflammatory phenotype. In this review, we delineate how bacterial pathogens both induce excessive NET formation, driving endothelial dysfunction, and evade NET-mediated antimicrobial defense. Here, we critically examine the signaling crosstalk between the NET formation with other cell death pathways, particularly focusing on the ROS-mediated PAD4-RIPK axis as well as the gasdermin-D mediated lytic signaling. Developing therapeutic strategies that target NET function presents significant challenges, requiring a balance between maintaining beneficial aspects and eliminating harmful effects. Given the rise in antimicrobial resistance, targeting host factors could lead to the development of more effective strategies. Together, this review explores the molecular mechanisms of NET formation, the paradoxical roles of NETs in different bacterial infections, their interplay with other cell death pathways, their impact on sepsis, and the exploration of different components of NETs as potential therapeutic candidates.

## 1 Introduction

Neutrophils are the most abundant cells and the first line of defense within the human innate immune system (1). Throughout their short lifespan in the bloodstream, they patrol for any signals corresponding to infections or alterations in homeostasis (2). Phagocytosis, degranulation as well as antibody dependent cell mediated cytotoxicity (ADCC) are the major protective mechanisms executed by neutrophils. Recently, the list has been expanded to include neutrophil extracellular traps (3). Fibrous web-like chromatin structures named NETs (4) are one among the wide range of antimicrobial mechanisms of neutrophils (5). They comprise decondensed DNA in association with histones and antimicrobials released from the granules present within (4). Volker Brinkmann, Zychlinsky and team has led to the discovery of this phenomenal mechanism in 2004 (6). Since then, the scientific research community has witnessed many novel mechanisms corresponding to NET release in various aspects. The increased number of results in PubMed when you search for “neutrophil extracellular traps” proves significant studies have been conducted on this topic. Twenty-two years later, our knowledge on NETs has increased significantly but remains an enigma. To form NETs, neutrophils must prepare and undergo several molecular processes. The fate of the neutrophil depends on the type of stimulus by which NET formation is activated. It could result in a dead or vital neutrophil at the end of the process, which will also be discussed in this review.

Pathological consequences of NET formation have been associated with many diseases, including autoimmune disorders, cancer, infections, and even diabetes (7). NETs have the potential to trap and kill pathogens; however, if not balanced, this could result in increased pathogenesis (8). Compromised function of NETs could potentially help pathogens evade the host system, creating a favorable niche for them. There would always be a question of concern that why would bacteria intensifies an immune defense mechanism by host. It is possible that under certain pathological conditions, some bacterial species could take advantage of triggering NET formation and employing these to compromise host tissues, overriding the antibacterial effects of NETs. This potentially benefits bacteria, thereby leading to efficient colonization and better access to the host’s resources, which could ensure an optimal survival advantage for the bacteria in the host (9). Neutrophils can also undergo other modes of cell death pathways as well. A comprehensive understanding of the relationship between NETosis and such pathways would also provide possible insights (10). In this review, we have also attempted to summarize the crosstalk between NETosis and major cell death pathways, including non-inflammatory apoptosis, over-inflammatory pyroptosis, and necroptosis. In connection to hyperinflammation and cell death, excessive NET formation or impaired clearance is considered a significant cause of sepsis (11), leading to a higher mortality rate. This could substantiate the need for better treatment strategies that target crucial NET markers.

Considering the relevance of the concept and the potential impact of this on society betterment, the future on more NET studies especially tracking on sepsis seem to be a need of the hour. Relevant literature was identified through searches of PubMed. Key search terms included “neutrophil extracellular traps”, “NETs”, “NETosis”, “PAD4”, “ESKAPE Pathogens”, and “sepsis”. Articles published between 2010 and 2026 were considered significantly along with the key landmark papers. Additional relevant studies were identified from the reference lists of key publications. As a narrative review, the search was intended to provide broad coverage of the relevant literature. In this review, we present a comprehensive understanding of the molecular mechanisms of NET formation, its association with various bacterial pathogens, the crosstalk between NETs and significant cell death pathways, and its role in sepsis, concluding with an overview of potential therapeutic targets against NETs.

## 2 Molecular architecture of neutrophil extracellular traps

NETs, are extracellular, filamentous structures released by activated neutrophils and are a major component of the innate immune defense. Their structural framework consists mainly of decondensed chromatin fibers (15–17 nm in diameter) which occupy about three to five times the volume of condensed chromatin and form the scaffold of the characteristic web-like architecture (1, 12). Nuclear DNA is the primary constituent of NETs, but mitochondrial DNA has also been observed in specific instances, pointing to the structural variability of these extracellular networks (8). This chromatin scaffold provides a platform for the entrapment of invading pathogens in a dense antimicrobial matrix, thus limiting microbial spread (13).

Proteomic analyses have shown NETs to consist of a complex array of nuclear, granular, and cytoplasmic proteins. Pioneering studies by Zychlinsky et al. identified 24 major proteins associated with PMA (phorbol 12-myristate 13-acetate)-induced NET formation, including histones, neutrophil elastase (NE), myeloperoxidase (MPO), cathelicidins, defensins, calprotectin and actin (8, 14). The overall protein composition is relatively conserved but quantitative differences in individual components have been observed depending on the nature of the activating stimulus (15). The variability in NET formation to different stimuli can impact the efficacy of NETs in killing antimicrobials and their immunomodulatory properties.

Histones represent almost 70% of the protein content of NETs and include the canonical histone subtypes H1, H2A, H2B, H3 and H4. Activated neutrophils have an increased abundance of histone H2 compared with resting neutrophils (16). Histones rich in arginine and lysine are extensively modified by post-translational modifications (PTMs), and among them, citrullination is a hallmark of NET formation (17). In NETosis, the enzyme peptidyl arginine deiminase 4 (PAD4) converts arginine residues to citrulline, leading to a decreased histone positive charge and supporting chromatin decondensation (18). Citrullinated histones are widely recognized as reliable markers for NET formation (19).

Azurophilic granules are one of the four classes of neutrophil granules and play a central role in NET-mediated antimicrobial activity. These granules release potent effector molecules, NE, MPO, cathepsin G, and defensins, that contribute to microbial killing after incorporation into the extracellular chromatin scaffold (20). The antimicrobial peptide LL-37 (cathelicidin) further stabilizes the NETs by forming resilient complexes with the extracellular DNA protecting the scaffold from degradation by the bacterial nuclease (21). Furthermore, LL-37 enhances macrophage phagocytic clearance of NETs and thus promotes the resolution of inflammation (22).

In addition to citrullination, NET-associated proteins are subject to other PTMs that regulate their biological activities. Hemmling et al. showed that the MPO activity-generated hypochlorous acid (HOCl) induces oxidative modifications of histones and other NET-associated proteins in the DNA scaffold (23). In addition, extracellular histones and other nuclear components can act as autoantigens and therefore contribute to the pathogenesis of antineutrophil cytoplasmic antibody (ANCA)-associated autoimmune diseases (24). NET-derived DNA, histones and granular proteins have also been shown to act as damage-associated molecular patterns (DAMPs) that activate pattern recognition receptors (PRR) and amplify inflammatory signaling (25). The complex molecular architecture of NETs underpins their antimicrobial activity and plays a role in immune regulation and immunopathology, suggesting that NET components are promising targets for therapeutic intervention.

## 3 Molecular mechanisms governing neutrophil extracellular trap formation

Different mechanisms of NET formation and the prerequisites for specific events to occur are discussed here. Lytic and non-lytic NET formation are the two fundamental models (26). The host and the triggering factor have an influence on which pathway to be executed (27). Neutrophils, being short-lived cells, make it challenging to perform genetic manipulation, which is a reason for the lack of sufficient exploration of the mechanism of NETosis (7). In contrast to the rapid processes of non-lytic NETosis, lytic NETosis takes more time, as it involves cell death (12). Although these broad mechanisms have been investigated by several groups worldwide, many unanswered questions remain regarding the intracellular processes within. This also reveals the research potential in this context.

### 3.1 Lytic NET formation

Lytic NETosis also known as Suicidal NETosis or NOX (NADPH oxidase)- dependent pathway has been known since the discovery of NET formation. The main triggers involved in this pathway are autoantibodies, cholesterol crystals, and chemical inducers, such as PMA (28). PMA has been observed to be the best inducer for studying lytic NETosis. Activation of surface receptors on neutrophils leads to the release of calcium ions from the channel within the endoplasmic reticulum (29). This leads to the activation of the RAF-MEK-ERK pathway (30) through associated protein kinase C activity (31). Activation of signaling pathways leads to the phosphorylation of the NOX complex, potentially by the synergistic activity of the aforementioned pathways and calcium ions (32). Reactive oxygen species (ROS) are produced in response to NOX complex activity (33). Increased levels of ROS could aid in the translocation of NE and MPO to the nucleus (13) by degrading the major azurophilic cytoplasmic granules of the neutrophil (29). In parallel to this, the calcium ions available in the cytoplasm does other activities as well. It activates a calcium-dependent enzyme known as PAD4. This enzyme converts arginine residues of the histone present in the nucleus to citrulline (34). This citrullination reduces the positive charge on histones, thereby weakening their interaction with DNA and resulting in decondensation of the chromatin (35). In 2009, just few years following the discovery of NETosis, Wang et al. have strongly proved the significant role of PAD4 where they observed that upon inhibition of PAD4, NET formation was disabled as well as its deficiency resulted in impaired NET formation (36). The decondensed chromatin associates with proteins present in the cytoplasm and is expelled via membrane lysis (12) with the help of cleaved gasdermin D, a pore-forming protein (29) which will be discussed further.

### 3.2 Non- lytic NET formation

Rather always resulting in cell death, neutrophils also undertake a different mode of NET formation termed non-lytic or vital NET formation, which is NOX-independent. Differences in the stimulus range are among the basic differences between the lytic and non-lytic pathways. The neutrophil surface receptors in this case gets triggered by stimuli such as gram-positive bacteria like *Staphylococcus aureus* or LPS from gram negative organisms (26). Similar to the lytic pathway, NE and MPO translocation occur here as well, along with PAD4 activity, but independent of NOX and ROS release (32). Conversely, vital NET formation necessitates the vesicular transport of decondensed chromatin from the nucleus to the extracellular milieu without compromising neutrophil membrane integrity (37). Special mention should be made of Pilsczek et al. for uncovering this pathway. They observed this unique response of neutrophils against *S. aureus*. Following the aforementioned mechanisms, multinucleated neutrophils rounded, and vesicles carrying decondensed chromatin budded off, ruptured, and released their contents extracellularly (38). It should be a question of concern that why should neutrophil undergo a non- suicidal pathway. It is interesting to note that even after releasing all nuclear contents, anucleated neutrophils continue their basic functions, such as capturing more pathogens, because their plasma membrane is not breached (37). Several arguments regarding the concept of normal functions by anuclear cells exist. However, strong investigations have proven the ability of anuclear cytoplasts, such as showing chemotactic signals and phagocytosing bacteria for the remaining period in their short lifespan (39).

**Figure 1:**
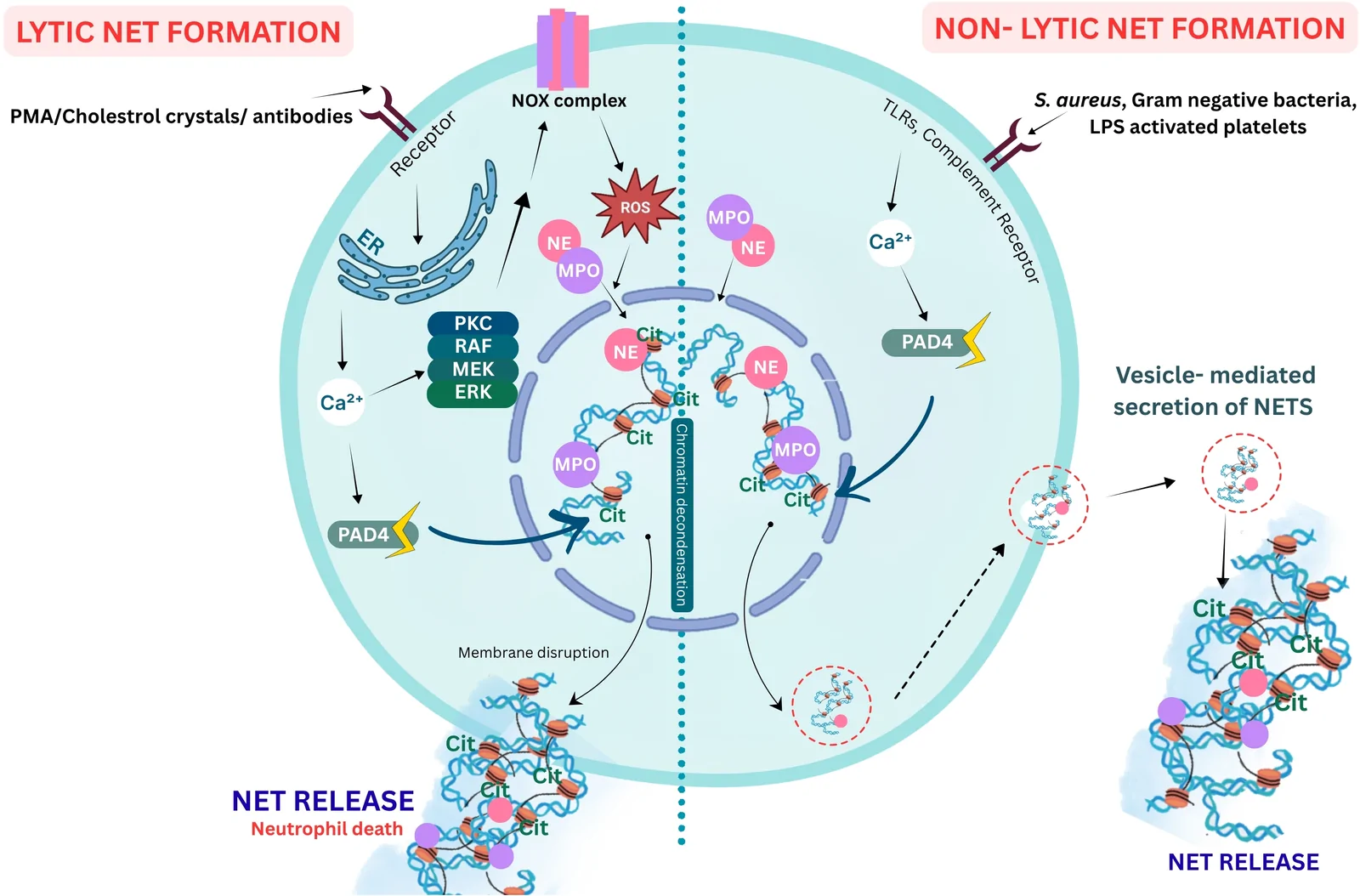
Molecular mechanisms of neutrophil extracellular traps. The two major mechanisms include lytic and non-lytic pathway. Upon stimulation, the neutrophils undergo signaling cascades either NOX dependent or independent. Activation and translocation of granules like neutrophil elastase and myeloperoxidase to the nucleus leads to chromatin decondensation. PAD4 enzyme plays a critical role in the citrullination of histones further facilitating the membrane disruption and the release of NETs. The image is created using Canva.

**Figure 1** schematically represents the characteristics of both pathways. The molecular characteristics of both mechanisms must be established further, which could increase our knowledge. Deciphering more information at the molecular level could potentially allow researchers to target specific components or pathways to balance this process. A more advanced level of interpretation is also needed, in addition to accepting normal assays as evidence. Although PAD4 mediated histone citrullination is a well-characterized mechanism for NET formation, it has been reported that NET release is not always dependent on PAD4 activity. Studies using different stimuli and experimental models have proved that NET release could occur in its absence as well, highlighting mechanistic heterogeneity in the pathways leading to the extracellular chromatin release (40). Therefore, it has to be understood that PAD4 dependent pathway is not exclusive, with the contribution of PAD4 and other signaling pathways varying according to the nature of the stimulus and cellular context. NETs also exhibit substantial structural heterogeneity, extending beyond the canonical diffuse chromatin structures to include aggregated NETs (aggNETs), in which extracellular chromatin and neutrophil-derived proteins form dense, enzymatically active aggregates (41, 42). This diversity suggests that NETs represent a spectrum of extracellular structures rather than a single uniform entity and should be considered when interpreting such functions.

## 4 Neutrophil heterogeneity and NET formation

Neutrophils are now recognized as a heterogenous population with distinct phenotypic and functional characteristics, rather than the traditional view of considering them as a uniform group. This diversity can arise from differences in maturation, activation, cellular aging, and tissue localization. These factors may influence their responses during infections (43, 44). This heterogeneity is significantly relevant to NET formation since different subpopulations possess different capacities to release NETs.

One key subunit is low-density granulocyte (LDG), which exhibits enhanced NET formation compared to normal-density neutrophils (45). During sepsis, the abundance of circulating LDGs increase where it comprises of cells at various developmental stages, highlighting that LDGs themselves as a heterogenous subpopulation (46). Therefore, the recruitment and activity of LDGs may influence NET-mediated inflammatory responses during sepsis.

Cellular aging further expands neutrophil diversity. Aged neutrophils are characterized by increased CXCR4 and reduced expression of CD62L. It can also display improved activation and NET formation under certain inflammatory conditions (47, 48). Furthermore, neutrophils recruited to different tissues can undergo local phenotype and functional adaptation in response to tissue microenvironment (49).

**Figure 2:**
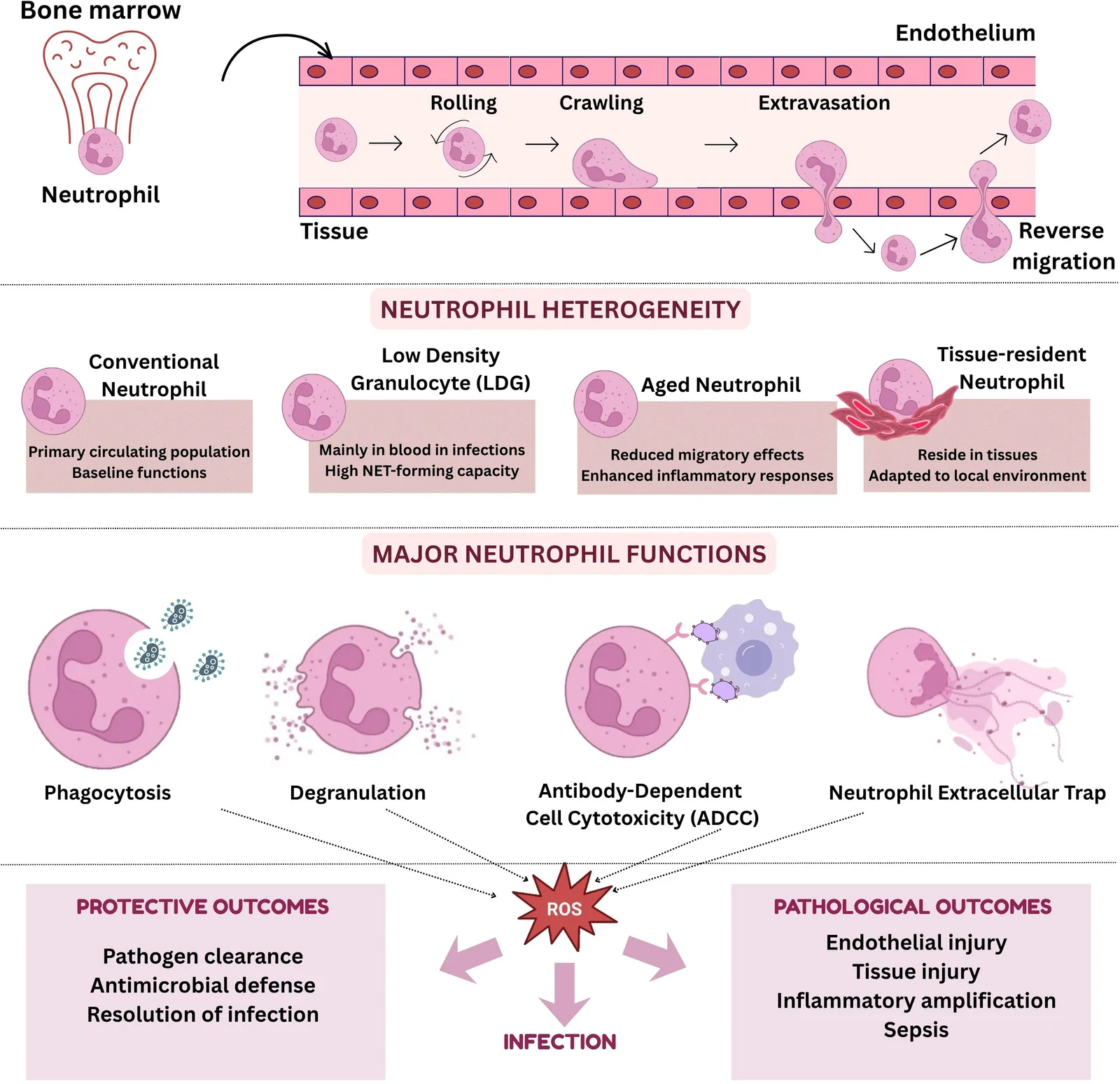
Neutrophil heterogeneity and functional plasticity in infection. Neutrophils undergo maturation in the bone marrow and diversify into functionally distinct states that shape their antimicrobial and inflammatory responses. These include phagocytosis, degranulation, NET formation, ADCC, and subsequent ROS generation, which can promote pathogen clearance or, when dysregulated, contribute to tissue injury, organ dysfunction, and sepsis. The image is created using Canva.

Overall, neutrophil heterogeneity should be considered when interpreting NET formation in infection and particularly sepsis. All these parameters can drive variations in NET formation and accounts for the broad spectrum of antimicrobial defense and host tissue damage associated with NETs (**Figure 2**).

## 5 Host–pathogen interplay: neutrophil extracellular traps in bacterial infections

NETs possess a crucial role in orchestrating immune defenses against bacterial infections. The dual nature of NETs could potentially affect the outcome of the disease. In control of infection, it could trap and even kill the pathogens whereas dysregulated NETosis could lead to chronic infection posing even more serious threats (50). Visual imaging evidence are available in many studies to prove the activity of neutrophils against various pathogens. Even if the host exhibits such sophisticated mechanisms, there are bacteria which have evolved to evade these. Here, we will be comprehensively discussing this context with the example of few bacteria.

The emerging antibiotic resistance and the declined antimicrobial pipeline are the major risks associated with public health. The ESKAPE pathogens, *Enterococcus faecium*, *Staphylococcus aureus*, *Klebsiella pneumoniae*, *Acinetobacter baumannii*, *Pseudomonas aeruginosa*, and *Enterobacter* spp, need a significant mention here. The urgent need of treatment against these pathogens was alarming us since decades. However, we remain in a situation where, with each passing year, the antibiotics effective against these pathogens face a progressive decline. With the knowledge that combating bacterial strategies is a futile task, we must learn from the past to preserve the treatment approaches (51).

Upon binding of pathogens to the DNA network, its adhesive properties and immobilizing characteristics trap the pathogen in an antimicrobial-concentrated milieu, potentially leading to its elimination and preventing further colonization (52). DNA can breach the microbial membrane and lyse the cells. Halverson et al. has proved that the application of excess cations and phosphatase enzyme neutralizes the antimicrobial activity of NETs as DNA requires the phosphodiester backbone to sequester the cations (53). The recognition of bacterial pathogens by neutrophils includes a range of pattern recognition and immune receptors. As a result, NET induction extends beyond the canonical TLR4 (Toll-like receptor 4) -NADPH oxidase axis. Along with TLRs, bacterial pathogens can be also sensed through NOD-like receptors (NLRs), C-type lectin receptors (CLRs), complement receptors, and Fc receptors, while protease activated receptor-2 (PAR-2) provides an additional route. Baz et al. has comprehensively mentioned some key pathogens contributing to these pathways. Group B *Streptococcus*, *Bordetella pertussis*, and *Porphyromonas gingivalis* have been associated with NET induction through distinct host-recognition pathways. These pathways can converge on downstream processes including ROS generation, calcium signaling, and inflammatory signaling (54).

*Enterococcus faecium, especially* vancomycin-resistant *E. faecium* (VRE), exhibits a significant ability to form biofilms on host tissues and indwelling medical devices contributing to reduced susceptibility to antimicrobial treatment. Extracellular DNA and other matrix components can alter neutrophil-bacterial interactions and provide a protected niche for bacteria (55). *S. aureus* has been a key organism in the history of NETosis. Brinkmann et al. in early 2000s, uncovered the major inferences of this new concept using *S. aureus* as the stimulus (6). Later, as we have already mentioned above, the same organism paved the way to reveal the vital NET formation (38). Upon infection, staphylococcal products are released, which could initiate the NETosis process. *S. aureus* could induce as well as neutralize the NETs according to its need. Its major factors that affect the antimicrobial activity of NETs include a nuclease that interferes with DNA, an extracellular adherence protein (EAP) that can stabilize DNA, and a surface protein, fibronectin-binding protein B (FnBPB), which acts against histones (9). At certain points, bacteria induce NETs to spread the disease. Certain proteins of *S. aureus*, such as Staphylococcal Protein A (SpA) (56), Leukocidin A/B (LukAB), Panton-Valentine Leukocidin (PVL) (57), Phenol soluble modulin α (PSM α) (9) are inducers. Using an isogenic nuclease-deficient mutant of *S. aureus*, Berends et al. have demonstrated that nuclease expression enables the pathogen to evade entrapment by NETs, thereby it enhances the resistance (58). Thammavongsa et al. also proved that *S. aureus* secretes potential enzymes which converts NETs into 2’- deoxyadenosine (dAdo) that could induce cell death leading to enhanced survival of the pathogen (59, 60). Notably, methicillin resistant *S. aureus* (MRSA) biofilms can actively manipulate NET responses like biofilm-associated leukocidins, including PVL and γ-haemolysin AB (61). These studies show that in MRSA, NET formation may coexist with bacterial persistence rather than necessarily resulting in bacterial clearance.

Wei Hung Lin and team reported that the uropathogenic *Escherichia coli* induces NET via the O-antigen mediated TLR4 pathway. Lipid A has traditionally been understood to mediate this pathway; however, the authors demonstrated the potential of O-antigen. It is interesting to observe that only a specific O-antigen can trigger this pathway; otherwise, it can even negatively modulate the pathway. The O-antigen has been shown to induce IL-1β, a NETosis amplifier, by host immune cells (62). In addition, some studies have shown that intravital microscopic analysis revealed that *E. coli* was seldom captured at the surface of neutrophils but rather entrapped in NETs even far from cells (63). TcpC or Toll/interleukin-1 receptor domain-containing protein C, a virulence factor of uropathogenic *E. coli*, degrades PAD-4 by ubiquitination, thereby inhibiting NETosis. This is a strong mechanism used by this pathogen to evade host immune defense (64).

*Klebsiella pneumoniae* liver abscess (KPLA) is associated with several sepsis-related conditions. In the previous year, Wang et al. published a study that revealed the impact of NETs on the progression of KPLA. They validated that *K. pneumoniae* utilizes the TLR4-PI3Kα-Akt pathway for the establishment of disease by over-activating NET formation in the host. NETosis driven by *K. pneumoniae* involving complement component C3 is the reason for endothelial dysfunction (65). Even if LPS of *K. pneumoniae* triggers the TLR4 receptor, Akt is a requisite for NETosis rather than apoptosis (66). An interesting study was conducted by Theodora A M Claushuis group to decipher the role of PAD4 enzyme upon infection with *K. pneumoniae* specifically in pneumonia-induced sepsis. Histone citrullination was observed in parallel with our understanding of the activity of PAD4 in converting arginine to citrulline. However, *K. pneumoniae* was able to result in NETosis even in the absence of the PAD4 enzyme, which was determined experimentally on Pad4^-/-^ neutrophils (18). With this work, the authors have opened an avenue of questions to readers, which broadens the potential of this concept in the scientific research field. This suggests that neutrophils may employ alternative mechanisms of chromatin decondensation. Thus, while PAD-4 represents an important regulatory component of NET formation, it should not be considered an obligatory mediator of all forms of NETs. In the continued search for an answer, we found another work by Sharma et al., where their results revealed a hitherto function of a receptor called Mincle. Mincle or Macrophage Inducible C-Type Lectin (67) receptors can regulate the autophagy pathway and lead to NETosis without ROS production during *K. pneumoniae* infection (68). Both *in vitro* and *in vivo* results proved the activity of Mincle in NET formation during pneumoseptic infection with *K. pneumoniae*. Mincle^-/-^ cells resulted in impaired NETosis, whereas treatment with tamoxifen, an autophagy inducer, recovered the NETosis process. Strain-specific modulations were also observed in the case of *K. pneumoniae. K. pneumoniae* ST258 has been reported to result in impaired NETosis by via affecting elastase mobility which inhibits the translocation of azurophilic granules like NE, MPO from cytoplasm to nucleus which is a key step in the NETosis pathway (69).

Due to the increased drug resistance rates, infections associated with *A. baumanii* has increased largely. Kamoshida and team are a strong group working intensely for deciphering the effect of *A. baumannii* upon NETs. They have observed that *A. baumannii* infected neutrophils were less phagocytic efficient making the pathogen easier to escape (70). They investigated the NET formation using *A. baumannii* as well as *P. aeruginosa* as stimuli. The pathogens were showing distinct characteristics upon infection on neutrophils. *P. aeruginosa* was found to induce NET formation whereas *A. baumannii* didn’t. Even though the pathogens share major virulence traits; their neutrophils’ response varies. The major finding of the study was the differential induction of antimicrobial granule release in response to these pathogens. The expression of NE and MPO was more in infection with *P. aeruginosa* than the other pathogen (71). In a follow-up study deciphering the host-pathogen interactions, the authors demonstrated that *A. baumannii* adheres to the surface of neutrophils and is subsequently transported by these migrating immune cells. With this mechanism of hijacking the neutrophils as dissemination vehicles, the pathogen spread to distant sites in the body. The authors described this phenomenon using the term “bacterial immunity taxi”, highlighting the ability of the pathogen to utilize the immune cell for dissemination (72). Two years later, the same group reported another intriguing finding, demonstrating that this pathogen increases the survival of neutrophils by preventing the formation of NETs. Since neutrophil adhesion is a significant requirement for the activation of neutrophils, *A. baumannii* was found to suppress the expression of CD11a (integrin αL), an important subunit of Lymphocyte Function- Associated Antigen-1 (LFA-1), in PMA-stimulated neutrophils (70). However, there are contradictory studies that have reported the NET formation induced by *A. baumannii* where the pathogen gets efficiently phagocytosed by neutrophils (70). The observed contradictory findings may be attributed to differences in experimental conditions, such as the bacterial strains, co-culture conditions, multiplicity of infection, and other variations. Therefore, further molecular and mechanistic studies are needed to draw more comprehensive understanding of the interactions between *A. baumannii* and neutrophils. P. aeruginosa as mentioned above is a significant example in this context. During biofilm-associated infection, neutrophils can form NETs at the biofilm interface, which may restrict bacterial dissemination but does not necessarily eradicate the established biofilm (73). The pathogen also carries strategies for directly counteracting NETs with secreted DNAses and phosphatase activities (74). Extracellular DNA can trigger neutrophil activation and NET release, creating a biofilm-associated inflammation and incomplete clearance of pathogen (75).

*Mycobacterium tuberculosis* is a very successful intracellular pathogen that carry many potential methods to defeat the immune defense mechanisms of the host. NETs could trap and contain the *M.tuberculosis* but fails to kill them (76). Chowdhury et al. recently have reported that the type 1 interferons are responsible for the NET formation during *M.tuberculosis* infection. They have observed that the interconnection of NET release along with necrosis and caseation can create an advantageous environment for *M.tuberculosis* to persist and multiply. Moreover, ensnaring *M.tuberculosis* without damaging during the NET release could subvert the host by promoting the survival of the pathogen (77)*. Pseudomonas aeruginosa*, a ubiquitous organism secretes a virulence factor called pyocyanin. This pigment could activate the NOX dependent pathway leading to NET formation (78). One another notorious bacteria is *Vibrio cholerae* where it induces NET formation by neutrophils as they are the first line of defense. Once the NET forms, the pathogen evades this via its two extracellular nucleases, Dns and Xds which degrades the extracellular DNA and helps in biofilm formation as well (79). *Salmonella typhimurium* was also a key bacterial model utilized by Brinkmann for the initial study of NETosis where it was observed getting ensnared and eliminated by the consolidated activity of NET components including histones and the granular proteins released (6). It is notable that how these bacteria have evolved to tackle a sophisticated immune defense mechanism by the host. **Table 1** summarizes the interplay between bacterial infections and NET formation. Even with the examples of these few bacteria, we should understand the potential of approaches that have to be developed against these bacterial strategies. Each organism exhibits different mode of action hence a deeper and complex understanding is necessary.

**Table 1:** Examples of bacterial pathogens and the mechanism of NETs involved.

Bacteria	Protein/pathway	Mechanism	Host outcome	References
*Staphylococcus aureus*	EAP FnBPB	Stabilizes DNA; acts against histones	NET degradation	(9)
*Staphylococcus aureus*	SpA LukAB PVL PSMα	Induces NET formation	NET induction	(56, 57, 59)
*Escherichia coli* (uropathogenic)	O-antigen	Releases IL1β which amplifies NET	Excessive NETosis	(62)
	TcpC	Degrades PAD-4 enzyme	NET degradation	(64)
*Klebsiella pneumoniae*	TLR4-PI3Kα-Akt	Overactivates NET	Excessive NETosis	(65)
	Via Mincle receptor	Activates NETosis via autophagy regulation	NET induction	(68)
*Klebsiella pneumoniae ST258*	Translocation of azurophilic granules	Impairs NETosis	NET evasion	(69)
*Acinetobacter baumannii*	Suppression of PMA-induced CD11a expression	Impairs neutrophil adhesion to other cells	NET evasion	(70)
*Pseudomonas aeruginosa*	Secreted phosphatase and DNase	DNase degrades extracellular DNA within NETs; Phosphatase contributes to bacterial resistance	Reduced NET-mediated bacterial killing	(74)
	Extracellular DNA	Stimulates neutrophil activation	Stimulates NET formation and limits bacterial clearance	(75)
*Mycobacterium tuberculosis*	Via Type 1 interferons	Induces NET	NET induction	(77)
*Pseudomonas aeruginosa*	Pyocyanin	Activates NOX dependent NET formation	Excessive NETosis	(78)
*Vibrio cholerae*	Dns Xds	Degrades DNA and helps in biofilm formation	NET degradation	(79)

## 6 Interplay between NETosis and programmed cell death pathways

Over the past few decades, cell death pathways have evolved from conventional active and passive pathways. It is significant to note the crosstalk between these pathways to understand how different mechanisms are executed by the host to attack pathogens. Moreover, pathogens are evolving at a higher rate to defeat this weapon or to take advantage of it.

### 6.1 NETosis and apoptosis

Apoptosis is a regulated, non-inflammatory cell death pathway that is dependent on the activity of various caspases (80). The downstream activity of these caspases results in notable features, including membrane blebbing, shrinkage of cells, fragmented DNA, and release of apoptotic bodies (81). Stimuli like DNA damage, metabolic stress conditions activate the intrinsic pathway of apoptosis. This results in the permeabilization of the mitochondrial membrane by pro-apoptotic proteins, such as Bcl-2 and Bax, thereby releasing cytochrome C (82). In contrast, extracellular factors, such as death receptors, trigger the extrinsic pathway of apoptosis (83). Disturbances in the apoptosis pathway may result in the activation of NETosis, although they work distinctly (84). We have previously mentioned the role of the Akt pathway in NETosis. It has been observed that Akt could act as an inhibitor of apoptosis (85). Immunofluorescence studies have revealed that upon treatment with Akt inhibitors, NETosis is reduced, whereas apoptosis is increased. This study shows that Akt is a molecular switch for apoptosis and NETosis (66). The same team later reported a beautiful concept known as ApoNetosis, a novel form of neutrophil death. Here they have demonstrated this using activity of UV. The characteristics differ from the normal process, which includes ROS release but is NOX-independent. They have also mentioned that in this form, there is no association of citrullinated histones, which could infer that this is even calcium ions and PAD4 independent (86). However more uncovering of mechanisms is needed in this aspect.

### 6.2 NETosis and necroptosis

Although apoptosis is a classic cell death pathway executed by the host, pathogens can sometimes easily inhibit it. Here comes the need of alternative pathways as part of immune defense by the hosts. Necroptosis is a programmed mode of necrosis that involves characteristic features, such as cell swelling, membrane rupture, and the release of contents within (87). Unlike the usual cell death pathways, necroptosis is caspase-independent. It is regulated mainly by receptor interacting protein kinase RIPK1 & RIPK2 (88) and Mixed lineage kinase domain- like protein MLKL, a pseudokinase (89). Caspase -8 inhibition results in phosphorylation events by RIPK proteins, thereby phosphorylating MLKL. Once activated, MLKL translocates to the plasma membrane and forms a pore, leading to cell lysis (87).

Schreiber et al. reported a link between necroptosis and NETosis in terms of ANCA. Their complementary experimental demonstrations showed that RIPK1&3 and MLKL strongly control ANCA-induced NET formation (90). They have strongly proven this *in vitro* and *in vivo* to an extent. In the case of necrotizing glomerulonephritis, renal biopsy immunostaining proved the presence of NET components, such as citrullinated histones, PAD4 enzyme, and MPO, accumulated around the fibrinoid necrosis (91). This shows the synergy between necroptosis and NETosis. Supporting these findings, D’cruz et al. reported that MLKL, upon activation, is followed by NET formation. They demonstrated this with strong phenotypic and functional characteristics, such as ROS production, translocation of neutrophilic granules, compromised nuclear membrane, and hyper citrullinated histones (92). This study unmasked significant signaling between the RIPK1-K3-MLKL axis and PAD4 activity, showing how necroptosis triggered NET release and the synergy controlled the infection caused by methicillin-resistant *S. aureus* (MRSA) (92). Patients with chronic granulomatous disease were found not to produce increased ROS because they have genetically defective NADPH oxidase. However, studies have proven that once activated, MLKL gains traction from RIPK proteins and thereby synergistically activates the PI3K pathway and NADPH oxidase (93). A better understanding in this context will aid in the treatment of many diseases.

### 6.3 NET and pyroptosis

Pyroptosis another programmed cell death pathway that is of an inflammatory nature (94). The basic mechanism involves a canonical and non-canonical pathway, which includes a consortium of molecules. Microbe-associated molecular patterns (MAMPs) trigger PRRs and activate inflammasomes primarily via nod-like receptors (NLRs). Once activated along with the subsequent trigger by DAMPs, procaspase-1 and Apoptosis- associated Speck-like protein containing CARD (ASC) components are recruited and they oligomerize to form the activated inflammasome. This converts procaspase-1 to caspase-1, which processes the pro-IL-1β, and pro-IL-18 into biologically active proinflammatory cytokines IL-1β and IL-18. In parallel to this, gasdermin D (GSDMD), an executioner protein of pyroptosis, gets activated and the released fragment goes and attaches to the membrane form pores, thereby releasing the pro-inflammatory cytokines (95).

**Figure 3:**
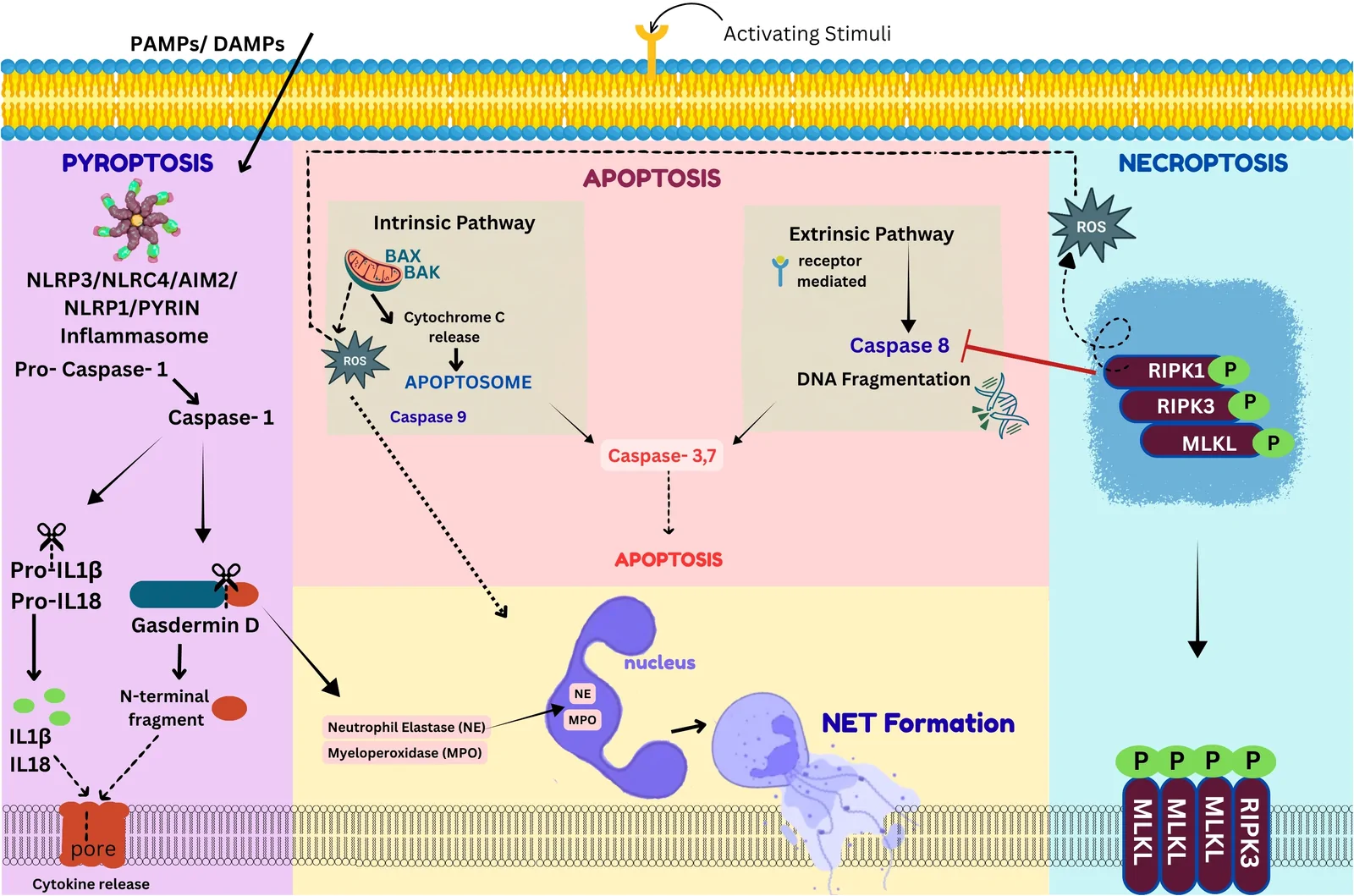
Crosstalk between NET formation and programmed cell death pathways. NETosis shares molecular components with apoptosis, pyroptosis and necroptosis. Inflammasome activation, caspase signaling, RIPK-MLKL mediated axis could intersect with NETosis. The image highlights the shared nodes among these pathways. The image is created using Canva.

Neutrophil pyroptosis can also be triggered by the abovementioned pathways. Studies have revealed that NET formation can intensify pyroptosis effects. Extracellular decondensed chromatin can augment pyroptosis through AIM2 inflammasome activation. Additionally, it has been reported that the azurophilic granule in neutrophils, NE, can activate GSDMD (96). In another study, it was reported that, in contrast to the activity of GSDMD in pyroptosis, its activity in neutrophils is caspase independent. A neutrophil elastase called ELANE cleaves GSDMD and releases its N-terminal fragment. However, this ELANE-mediated fragment is as efficient as the classic gasdermin fragment (97). Linsong Chen and team reported that High Mobility Group Box 1 (HMGB1) associated with NET ejection could aid in the pyroptotic death of macrophages. HMGB1 is responsible for many cellular functions, such as repairing damaged DNA and immune responses (98). Here, HMGB1 is a DAMP that triggers the NF-κB pathway and intensifies the secretion of interferons and pro-inflammatory cytokines (99). These findings suggest that GSDMD should not be considered solely a pyroptosis effector but also a mechanistic link between inflammasome activation, NETosis, and immunothrombosis. Although many studies have focused on the crosstalk between NETs and pyroptosis, it is essential to conduct more investigations to gain a better understanding of the molecular level interactions between these pathways. **Figure 3** schematically represents the interplay between NET formation and the aforementioned cell death pathways.

## 7 Neutrophil extracellular traps in sepsis: balancing host defense and immunopathology

Sepsis is a life-threatening disease syndrome characterized by severe abnormalities due to impaired organ function (100). Kristina et al. reported the global incidence and mortality rate of sepsis over a decade, which serves as a landmark study (101). Their findings showed that approximately 48.5 million cases of sepsis and 11 million sepsis-associated deaths were reported in 2017, which is double that of previous years (101). Sepsis is associated with a diverse range of immune responses resulting from pathogenic triggers, such as MAMPs, and host triggers, such as DAMPs (102). Dysregulation of inflammatory pathways is the reason for the occurrence of sepsis (103). Excessive death of immune cells is the major reason for this (104). Excessive infiltration of neutrophils could damage the tissues which will be associated with excessive ROS production, granular protein release as well as NET formation (5). The transition of the anti-inflammatory to pro-inflammatory phenotype in endothelial cells due to NET formation can result in glycocalyx degradation, which increases cell permeability (105). A group of researchers demonstrated that patients with pulmonary hypertension showed elevated levels of NET markers. The increased levels were through MPO/H_2_O_2_ associated activation of TLR4/NFκB pathway (106). Chao Song et al. established the increased M1 phenotype of macrophages induced by the NET formation. As a result, increased release of pro-inflammatory cytokines, including IL-1, IL-6, and TNF, as well as the chemokine CXCL8 (formerly IL-8), was observed. This cytokine storm is a key cause of worsening of the condition into sepsis (107).

An interesting study by McDonald et al. in 2012 demonstrated a unique mode of action by neutrophils during sepsis conditions. Acute sepsis often leads to the presence of activated neutrophils in liver sinusoids. It is believed that this pathology is due to the hijacking of the host system by bacteria for their advantage. Their evidence proved that this recruitment is a host defense strategy, as liver sinusoids are also a niche for many Kupffer cells. These activated neutrophils can form and release NETs, which can effectively ensnare bacteria and prevent dissemination (63). Their previous studies have also proven the impact of adhesion by the interaction between the surface receptor CD44 and hyaluronan, leading to reversible adhesion of neutrophils. Refraining from this interaction led to reduced pathological consequences in the liver (108). The molecular mechanism varies from one condition to another. Considering the effect of histones on organ dysfunction, researchers have investigated sepsis in PAD4^-/-^ mice. No significant difference was observed in the knockout and wild-type strains upon polymicrobial sepsis (109).

Although NETs are considered to contain infection, they can sometimes extend to exacerbated levels, leading to damage in host tissues. The double-edged sword characteristics of NET formation have been well established (110). One of the common and severe outcomes of sepsis is acute lung injury (ALI). Lefrancais et al. in 2018 reported higher levels of NET formation in both the case of ALI as well as bacterial pneumoniae conditions (111, 112). A clinical study proposed by Margraf et al. more than a decade ago showed a correlation between the levels of various NET markers and sepsis scores (113). Within a few years of the discovery of NETs, Clark et al. revealed the antimicrobial activity of neutrophils by bacterial clearance via NET formation. This is mostly at the expense of tissue injury, ultimately leading to sepsis and septic shock. They demonstrated the role of TLR4-mediated platelet activation, resulting in NETosis and severe sepsis (114). Terence Bukong and team have investigated the role of NETs in patients with acute sepsis. Their studies on mouse models led to the observation that binge alcohol consumption induced impulsive formation of NETs and impaired efferocytosis. As efferocytosis is dysregulated, it affects the clearance of NETs, which ultimately results in systemic inflammation and lung injury (50).

S100A8/A9 is a key trigger of NET formation in sepsis conditions. As a DAMP, S100A8/A9 interacts with PRRs, including TLR-4 and the receptor for advanced glycation end products, thereby amplifies the pro-inflammatory signaling pathways promoting NETosis. The resulting NETs further stimulates the release of S100A8/A9, establishing a self-amplifying positive feedback loop that exacerbates inflammation, endothelial dysfunction, and tissue injury during sepsis. It is considered to interact with PRRs then amplify the inflammatory signaling which thereby triggers NETosis as a positive feedback loop (115, 116). NETs can subsequently function as prothrombotic scaffolds that facilitate platelet activation and coagulation, thereby contributing to microvascular thrombosis and sepsis-induced coagulopathy (117). Importantly, emerging clinical evidence has showed that GSDMD and NET associated biomarkers are elevated in patients with sepsis-induced coagulopathy, with N-GSDMD and MPO circulating, showing associations with glycocalyx injury and adverse clinical outcomes (118).

All these reveal growing evidence that pathogens are not only responsible for sepsis but also the hyperactivated immune system of the host. Uncovering more molecular mechanisms involved clinically would strengthen the claims to resolve many unanswered questions regarding the association with NETs and sepsis.

## 8 Targeting neutrophil extracellular traps: emerging therapeutic approaches

Considering the pathological activity of excess NETs, many research labs around the globe is investigating on various measures to treat this. More targeted strategies are needed to augment better clearance of NETs released (119). Various components involved in NETosis could serve as potential therapeutic targets to degrade or control NET formation.

Nucleases are one among the major anti-NET strategy since DNA being the structural backbone. As discussed above the accumulation of activated neutrophils without clearance of NETs could cause significant damage to the host tissues. Inhalation of DNase 1 has been observed to prevent the accumulation of these in the lungs of mice with transfusion- related acute lung injury (TRALI) (120). Clearance of NETs using DNases is a gold strategy to target NETs in case of excess activity. However, considering pathogens which produce nucleases itself to suppress the host defenses could achieve a survival advantage. Genes encoding DNases were observed in group A Streptococcus (GAS) which promoted the NET degradation and viability of the pathogen. They have validated this activity using a DNase blocker, G-actin which resulted in better clearance of pathogen by neutrophils (121). Similarly, *Mycobacterium bovis* produces MnuA, another nuclease which could potentially degrade NETs (122). Likewise, many pathogens do produce different nucleases for this purpose, and which shows us the significance of developing specific drugs. As discussed above, *A. baumannii* utilizes various strategies to evade NET formation. A study has demonstrated that clarithromycin, a macrolide antibiotic, enhances NET formation in neutrophils during the *A. baumannii* infection. This effect was shown to be mediated through the induction of autophagy and the decoration of NETs with LL37, an antimicrobial peptide. Interestingly, it was observed that clarithromycin-induced NETs, enriched with LL37, exhibited enhanced antimicrobial activity by suppressing bacterial growth as well as biofilm formation (123).

Yongqing et al. established the activity of two inhibitors associated with NET formation. The inhibitors include suberoylanilide hydroxamic acid (SAHA), which is a histone deacetylase inhibitor and Cl-amidine, which is a pan- PAD (Peptidyl arginine deiminase) inhibitor. Both inhibitors blocked the citrullination process which significantly improved the survival of septic induced mice models. They were the first to declare that citrullinated histones could serve as a potential target to treat sepsis besides being a NET marker (124). PAD-4 has emerged as an important pharmacological target for reducing NET formation. GSK484 is one another PAD-4 inhibitor which potentially showed reduction in thrombus formation (125). Earlier studies were mostly focusing on irreversible inhibitors like Cl-amidine and reversible inhibitors like GSK484. More recently, structure guided approaches have identified reversible, non-covalent allosteric inhibitors. The major target of such inhibitors is a calcium-dependent regulatory site distinct from the canonical catalytic pocket. These inhibitors stabilize an inactive, calcium-deficient conformation of PAD4 thereby suppresses citrullination in neutrophils (126). These advances expand the repertoire of PAD4-directed pharmacological strategies highlighting allosteric inhibition, a promising approach in NET-mediated diseases (126). Early proof-of-concept studies demonstrated that citrullinated histone H3 (CitH3) could serve as a potential therapeutic target in sepsis. This provided initial evidence that neutralization of this could reduce the detrimental effects of inflammatory pathways associated with NET formation (124). Building on these findings, a significant study by Ouyang et al. has demonstrated an emerging therapeutic strategy for targeting NET-associated pathological conditions, using humanized monoclonal antibodies against CitH3. Unlike approaches that broadly inhibit the NET formation process, anti-CitH3 antibodies aim to neutralize the downstream inflammatory effects of extracellular CitH3 without damaging the antimicrobial functions. The authors established reduced NET formation, inflammatory cytokine secretions, tissue damage, and moreover enhanced survival in experimental models of endotoxemia and bacterial sepsis. Favorable pharmacokinetic profiles and toxicology studies in rodents as well as non-human primates further supported the translational potential (127). This can be considered one of the advanced preclinical NET- targeted therapeutic strategies that warrants clinical evaluations. Studies on just PAD-4 inhibitors may not suffice the requirement. Mitochondrial DNA being un-associated with histones may require other specific inhibitor treatments (83). Besides these, platelet inhibitors also do the inhibitory activities against NETs. Treatment with clopidrogel, a platelet inhibitor, on mice with renal ischemia reperfusion injury, decreased the activity of NETs (128). A selective neutrophil elastase inhibitor called sivelestat is known to treat acute lung injury. In nephritic rats, elevated levels of proteinuria were observed which then reduced upon treatment with this inhibitor (129).

Recombinant human thrombomodulin was observed to attenuate coagulopathy in septic rats (130). Hydroxy ethyl starch treatment resulted in suppressed attachment between neutrophils and platelets. It also reduces the extravasation of neutrophils (131). Disulfiram, a gasdermin-D specific inhibitor was observed to reduce NET formation and suppresses inflammatory injury (132). Exenatide, a glycemic control agent was examined and found that it suppresses the ROS levels which subsequently reduces NET formation (133).

**Table 2** summarizes the potential compounds available to target NET at various disease conditions. A major challenge in developing therapeutics against NETs is to specifically target only the excessive NET formation without compromising the host’s immune system. Therefore, it is essential to develop therapeutics to maintain the regulated control of NETs. The approach to NET-targeted therapy will vary depending on the disease involved. With the vast amount of evidence from various disease conditions, we could understand that some requires the clearance of NETs while others benefit from the activity of NETs. The key is to achieve a balance. The variability of NETs, the off-target effects by the inhibitors, the possibility of compromising the host immune defense mechanisms are all risks associated.

**Table 2:** Potential compounds targeting NET formation.

Compound	Mechanism	*In vitro*	Pre-clinical/*in vivo*	Clinical phase	Current development status	References
Inhalation of DNase 1	Prevents accumulation of NETs in lungs of mice with TRALI	Yes	Yes	Phase 2	Clinical investigation: not approved for NET-targeted indication	(120)
Clarithromycin	Prevents A. baumannii growth and biofilm by enriched LL37 neutrophils in autophagy dependent manner	Yes	Yes	No specific NET-targeted trial identified	Approved drug but preclinical fro NET modulatory effects	(123)
SAHA	Histone deacetylase inhibitor	Yes	Yes	Phase 1–3 for oncology	Approved drug but NET application remains preclinical	(124)
Cl-amidine	Irreversible Pan-PAD inhibitor	Yes	Yes	No	Preclinical PAD4 inhibitor	(124)
GSK484	Reversible PAD-4 inhibition	Yes	Yes	No	Preclinical PAD4 inhibitor	(125)
Reversible allosteric PAD-4 inhibitors	Non-covalent inhibition of PAD-4 via calcium dependent allosteric regulatory site	Yes	Yes	No	Preclinical stage	(126)
Humanized anti- CitH3 monoclonal antibody	Neutralizes extracellular CitH3, reducing CitH3-driven NET formation and inflammatory signaling	Yes	Yes	No	Preclinical biologic	(127)
Clopidrogel	Platelet inhibitor	Yes	Yes	No	Approved antiplatelet but NET application remains experimental	(128)
Sivelestat	Neutrophil elastase inhibitor	Yes	Yes	Phase 2/3-type clinical evidence	Clinically investigated but not approved for NET-targeted applications	(129)
Recombinant human thrombomodulin	Attenuates coagulopathy	Yes	Yes	Phase 2-3	Clinical stage but NET application remains experimental	(130)
Hydroxyethyl starch	Reduces attachment of platelets and neutrophils	Yes	Yes	No NET-targeted study	NET application is preclinical	(131)
Disulfiram	Inhibits gasdermin-D activity	Yes	Yes	Yes, but not as GSDMD inhibitor	Approved drug but GSDMD targeted application remains preclinical	(132)
Exenatide	Glycemic control agent	Yes	Yes	Phase 3 approved but not as NET inhibitor	Approved GLP-1 receptor agonist: NET application remains preclinical	(133)

## 9 Discussion

NETs and their complex mechanisms have been of interest since Brinkmann et al. discovered them. Later, the gradual uncovering of its detrimental factors became more attractive. The revelation of the double-edged sword characteristics of NETs is a breakthrough for the development of various therapeutic strategies. NET formation is highly context-dependent. The dose of the stimuli, as well as the time of release and sustainability, matter. The release and clearance of NETs at the appropriate time regulate this process. Much potential exists for expanding our knowledge in this area with the emerging discipline of NETOMICS, which employs proteomic and genomic methods to analyze the molecular makeup of released NETs (7). Whole-blood genomic profiling studies have been in progress for many years. However, the rise of single-cell RNA transcriptomic studies has broadened the potential of detecting rare cells present in the pool (134). The significance of machine learning, especially in sepsis-related conditions, should be mentioned here. Machine learning is a potential tool to evaluate high-dimensional data (135) and to generate prediction tools (136), demonstrating the considerable promise of this discipline. The integration of artificial intelligence with multi-omics studies represents a transformative advancement, particularly in developing precision medicines against sepsis. The additive effects of both techniques together could revolutionize research and clinical investigations (137).

Bacteria possess a wide range of mechanisms to hijack the host immune defense system. The current marathon of research in this could hopefully untangle more pathways and molecules involved. As previously mentioned, the effects of NETs on bacteria have mainly been studied under *in vitro* conditions. The limited *in vivo* research is a drawback to be noted. The need for specialized technical knowledge could be difficult when considering *in vivo* studies on NETs. The optimization of different parameters associated with NETs, including the inducers, dosage, and duration for each component, is difficult to achieve *in vivo*. Researchers are currently at the infancy stage of exploiting genetically modified animal models to obtain a more advanced understanding of the role of specific genes, proteins, or signaling pathways involved in the regulation of NET formation (54). The available literature on this topic is exponentially increasing as we move forward. Considering the increased interest, many questions remain to be resolved. A deeper and more comprehensive understanding of the beneficial and detrimental effects of NETs would help us to exploit this host phenomenon for better treatment strategies. However, translating inhibitors from the laboratory to the bedside may take more time, which is the most important factor to consider.
